# Social Capital in Care for Complex Patients: A Pilot Study to Develop an Instrument for Measuring the Impact of Social Capital in Healthcare in Croatia

**DOI:** 10.3390/healthcare13131570

**Published:** 2025-06-30

**Authors:** Maja Banadinović, Marko Marelić, Dorja Vočanec, Aleksandar Džakula

**Affiliations:** 1 Center for Health Systems, Policies and Diplomacy, “Andrija Štampar” School of Public Health, School of Medicine, University of Zagreb, 10 000 Zagreb, Croatia; dvocanec@snz.hr (D.V.); adzakula@snz.hr (A.D.); 2 Department of Medical Sociology and Health Economics, “Andrija Štampar” School of Public Health, School of Medicine, University of Zagreb, 10 000 Zagreb, Croatia; marko.marelic@snz.hr

**Keywords:** social capital, care for complex patients, professionals, position generator

## Abstract

Background: The increasing complexity of patient care demands coordinated and integrated approaches involving multiple health and social care professionals. Social capital among professionals plays a critical role in facilitating effective collaboration and continuity of care for complex patients. Objectives: This pilot study aimed to develop and test an instrument based on the position generator method to measure perceived, potential, and activated social capital for patients within professional networks involved in complex patient care in Croatia. Methods: The instrument enabled differentiation between the existence of professional connections and their actual mobilization for patient benefit. This multidimensional approach, including activation levels for patient needs, marks a key improvement over earlier measures. Results: Results indicated that while professionals possess broad networks, the activation of these networks for patient care remains limited. Accumulated work experience is positively associated with greater activation of social capital, whereas formal changes in the work environment showed no significant impact. Conclusions: Despite sample limitations restricting generalizability, the instrument demonstrated sensitivity and applicability for mapping professional networks in healthcare settings. This study lays the groundwork for further research with larger samples to validate the instrument and support the development of coordinated care systems leveraging social capital for improved outcomes in complex patient care.

## 1. Introduction

In recent years, health systems across Europe have faced growing pressure to move beyond the traditional biomedical model and adopt a biopsychosocial approach that considers health as more than just the absence of disease. This perspective, which includes physical, mental, and social well-being, is increasingly recognized as essential for addressing the complex needs of contemporary patient populations [1]. Despite this paradigm shift, many healthcare systems remain primarily focused on medical treatments and technological solutions, often neglecting the broader social determinants that crucially shape health outcomes [2].

This gap is especially pronounced in the care of complex patients, individuals whose needs extend far beyond a single diagnosis and require highly individualized, coordinated care that cannot be met through standard care protocols or routine interventions. Complex patients are typically characterized by multimorbidity, significant functional limitations, and the presence of socioeconomic or behavioral factors that further complicate access to and provision of care. As such, complexity is understood as a multifactorial construct that may arise from medical, functional, psychological, or social causes. These dimensions are not mutually exclusive and often coexist, increasing the need for coordinated, interdisciplinary care. Consequently, these patients are often referred to as patients who “need help to receive help”, making them highly dependent on system support and intersectoral collaboration [3,4,5].

The concept of the complex patient has gained prominence in both research and policy discourse, particularly in the context of ageing populations, rising multimorbidity, and the increasing demand for long-term and integrated care [6,7]. In Croatia, as in many European countries, these trends are placing significant strain on both health and social care systems. International organizations such as the WHO emphasize the need to rebuild and strengthen health and long-term care systems and to foster integration and coordination across sectors to address the multifaceted needs of complex patients [8]. Complex patients thus represent not only a medical concern, but also a broader systemic challenge that extends to social services, civil society, and the community at large. Effective care for complex patients demands coordinated, intersectoral collaboration to ensure timely, integrated, and sustainable support [4,7,9].

In response to these challenges, Croatia has introduced key roles to improve care coordination, such as palliative care coordinators in primary care and hospital-based discharge planning services. These roles serve as essential connectors between institutions, professionals, and care levels, facilitating both horizontal and vertical integration across sectors. Palliative care coordinators focus on connecting community-based stakeholders to provide ongoing support, while hospital discharge services smooth the transition from hospital to primary or home-based care [10]. While general practitioners, community nurses, and other professionals remain essential in caring for complex patients, these coordinating roles help prevent over-reliance on any single provider and support a multidisciplinary approach [11].

Aligned with broader European trends toward strengthening long-term care systems [8,12,13], Croatia has made notable legislative progress. The National Health Development Plan of the Republic of Croatia 2021–2027 explicitly recognizes the need for an integrated model of health and social long-term care, based on a 24/7/365 approach [14]. The Health Care Act has introduced the concepts of the “complex patient” and “long-term care”, establishing a legal framework for developing new organizational models tailored to meet the specific needs of this population [15]. The 2023 Plan and Programme of Health Care Measures further operationalize these goals by identifying and defining subgroups of complex patients based on their care needs [16].

Despite these advancements, professionals involved in the care of complex patients still face significant challenges in coordination and intersectoral collaboration. Care systems often operate in silos, with limited communication and coordination between health, social, and community services [17]. As a result, patients are frequently left to navigate fragmented systems on their own. Where professionals are willing to take on this role, the necessary conditions for seamless information flow and care plan implementation are often lacking: there is a lack of IT system integration, limited awareness of available resources within the system, no allocated time, and no compensation for this type of work, etc. Although the number of professionals involved in patient care has increased significantly [18,19], little has been done to operationalize professional networks, not only in terms of establishing structural frameworks but also in fostering the necessary mindset and providing education to professionals for effective information exchange aimed at improving care quality and clinical outcomes. In practice, many rely on personal networks and informal contacts to bridge these gaps and ensure timely care, especially in urgent or complex cases. For example, a hospital-based nurse may urgently contact a trusted colleague to secure home nursing or orthopedic aids for a bedridden patient, leveraging their professional network to ensure continuity of care. These informal resources built over years of professional experience are commonly referred to as social capital.

Social capital, as described by Field, refers to the connectedness within networks where shared values are exchanged, becoming a valuable resource and, ultimately, a form of capital [20]. Different theoretical approaches to social capital exist, making it challenging for researchers to reach consensus, as various disciplines emphasize different dimensions and mechanisms [21]. Putnam defines social capital as features of social organization, such as trust and norms, that improve the efficiency of society by facilitating coordinated actions [22]. He distinguishes between bonding social capital, which links people within similar groups, and bridging social capital, which connects individuals across different groups, professions, or sectors and enables broader cooperation [21]. In the context of complex patient care, bridging social capital plays a particularly important role, acting as the sociological “WD-40” of the system, facilitating collaboration, reducing delays, and helping diverse professionals navigate a fragmented environment [23]. By enabling faster, more effective cooperation between diverse stakeholders, this form of social capital contributes directly to timely, coordinated care, especially in systems where formal structures are insufficient or under strain.

While the use of social capital may appear as a positive characteristic reflecting the resourcefulness and capability of professionals, from the perspective of system sustainability, it can also present a challenge. Relying on informal networks and personal contacts helps bridge existing gaps in the system, but in the long term, it further highlights structural weaknesses and makes the system dependent on individuals and their personal connections. In a broader context, social capital is often perceived negatively, as a mechanism for favoritism, privileged connections, or nepotism, particularly when used for personal gain [24]. However, in the case of complex patient care, professionals typically use their personal social capital for the benefit of patients, ensuring timely and appropriate care. This form of capital, while often invisible, represents a key component of the sustainability of the care system. In this study, social capital is conceptualized in a broader sense, not limited to family relations or personal social support, but focused on professional, institutional, and intersectoral networks that enable cooperation across organizational boundaries in the delivery of care. However, when such networks remain informal and individually dependent, the system becomes vulnerable to disruption, particularly when key professionals leave the system due to retirement or job change, taking their networks of contacts with them.

Quantifying social capital is essential for understanding its impact and limitations within healthcare systems. While studies confirm that social capital can improve access and continuity of care, excessive reliance on informal networks may increase inequalities and system vulnerability, especially for those lacking such connections [23]. Several instruments exist for measuring social capital; however, they primarily focus on internal organizational workplace dynamics [25,26,27]. Tools that capture the multidimensional and intersectoral activation of social capital, particularly how professionals activate and use it in the care of complex patients, remain limited [9,28]. There remains a clear need for the development of tools that capture how social capital operates in the complex care of patients, tools that can inform systemic interventions and reduce dependency on individual networks.

For the purposes of this study, perceived social capital refers to the presence of personal contacts across professional roles, regardless of their strength or use. Potential social capital denotes the respondent’s belief that these contacts could be mobilized to request help or support if needed. Activated social capital for the patient represents the actual use of such contacts within the past year to assist a patient. These distinctions allow for a more nuanced understanding of how professional networks function, not only in terms of existence, but also in their practical value for care coordination.

The general objective of this research is to examine the extent to which professionals activate and use their social capital in the care of complex patients.

The specific objectives are as follows:

- To develop and test an instrument for measuring perceived, potential, and activated social capital among professionals involved in the care of complex patients.
- To measure and compare the three forms of social capital according to gender, years of work experience, and the number of workplaces of the included professionals.

## 2. Materials and Methods

### 2.1. Research Background and Instrument Development

This pilot study was conducted within the Center for Health Systems, Policies and Diplomacy at the School of Medicine, University of Zagreb, as part of the program “Improving Care for Complex Patients”, which is focused on the development of integrated and long-term care with a specific emphasis on complex patients since 2022. Through research and professional activities conducted within the program, it was recognized that professionals involved in the care of complex patients often rely on their personal networks and informal contacts to ensure appropriate care for patients and their families. This form of informal networking points to the presence and importance of social capital as a key, yet insufficiently recognized, resource within the system. The social capital of professionals represents an invisible resource that contributes to system connectivity, especially in the context of providing care for complex patients, whose care requires a high degree of multidisciplinary approach and collaboration. Based on these observations, this pilot study was initiated to methodologically capture and evaluate the social capital of professionals in the model of care for complex patients, where the use of personal social networks is particularly emphasized to ensure effective and coordinated care.

### 2.2. Instrument Design

To measure the social capital of professionals, the position generator method was used [29], which enables the mapping of respondents’ social networks through the identification of acquaintances based on a predefined list of occupations. In this study, a new instrument was developed that includes a list of occupations key to the coordination, organization, and provision of care for people with complex needs at various levels of the system. The list included occupations such as physicians of various specialties, nurses, social workers, occupational therapists, pharmacists, representatives of non-governmental organizations and local government, and other relevant professionals. The criterion for selecting occupations was their direct or indirect involvement in coordinating, supporting, or providing care to complex patients. A detailed list of occupations is available in the survey questionnaire found in Appendix A.

The instrument is an integral part of a broader survey questionnaire consisting of three parts. In the introductory section, participants were informed about the purpose and aim of the research, the anonymity of the collected data, and the voluntary nature of participation. In addition, informed consent was included, through which participants gave their consent to participate in the research.

The first part of the survey questionnaire focused on measuring three forms of social capital among professionals, through a question in which participants were asked to indicate (1) whether they have personal contact with people from the listed occupations (perceived social capital), (2) whether, based on that contact, they can request help or a service for themselves (potential social capital), and (3) whether, through these acquaintances, in the past year they have requested help or a service for their patient (activated social capital for the patient). The three forms of social capital were measured to distinguish whether professionals have personal contacts, how strong these ties are, and whether they have used them for the benefit of the patient. Specifically, during the work of the Center, it was observed that professionals often rely on personal contacts to speed up or facilitate access to services for their patients. This prompted the need for a deeper insight into the difference between mere connectedness and the actual activation of these ties in everyday work. To make this possible, it was necessary to go a step further than the usual application of the position generator method, which primarily focuses on mapping the presence of acquaintances. The method was expanded to include an assessment of the strength of these ties, i.e., the possibility of using them for personal needs, as well as their actual activation for the purpose of helping patients in the past year. The purpose of this was to distinguish the mere existence of a tie from a real tie that can be used for a service or help. Since professionals, by the nature of their work, establish a large number of acquaintances, a high level of connectedness is expected. However, the mere fact that they know someone does not necessarily mean that they can activate that tie. Acquaintances from whom it is possible to request help for a patient suggest a greater strength of these ties, which in the context of social capital is often described as the difference between strong and weak ties.

In the second part of the survey questionnaire, respondents, using the instrument containing the same list of occupations as in the first part, ranked the listed positions according to the challenge of using personal contacts to obtain help or services for patients. Each respondent received an initial order in random sequence, and then used the drag-and-drop option to arrange the order according to their own opinion. Higher-ranked occupations were perceived as those with greater influence or power within the system.

The third part of the survey questionnaire included basic demographic data of the participants, including gender, years of work experience, profession, and the number of workplaces they have changed during their career. The selected variables were used to test the newly developed indices. The research was conducted via the online platform SurveyMonkey, and respondents accessed the questionnaire via their mobile devices.

### 2.3. Participants

The study sample comprised participants of the workshop held during the professional conference “Improving care for complex patients”, which took place from 30 May to 1 June 2024, in Biograd na Moru, Republic of Croatia. It was organized by the Center for Health Systems, Policies and Diplomacy at the School of Medicine, University of Zagreb. The conference was designed as a professional platform for exchanging knowledge and experiences related to the challenges and opportunities in integrating health and social care, with a strong focus on intersectoral coordination and collaboration in managing complex patients [30,31].

The aim of the conference was to assess the current state of long-term care and to develop recommendations for the preparation of strategic documents in the field of long-term care in the Republic of Croatia. The program and discussions were aligned with the National Strategic Plan for the Development of Healthcare 2021–2027 [14] and the World Bank’s policy brief on long-term care challenges and opportunities [32], ensuring that the dialogue was grounded in both national priorities and international evidence-based frameworks.

The participants were professionals from diverse backgrounds with experience in the organization, provision, and coordination of long-term and integrated care for complex patients. According to the structure, the participants represented both the healthcare and social welfare systems. Among the participants active in the healthcare system were professionals with formally defined coordination roles, such as discharge planning nurses and palliative care coordinators. In addition, there were other professionals who do not hold a formally recognized coordination role but whose daily work requires active involvement in the planning and delivery of care for complex patients, including staff from health centers, emergency services, and home care institutions.

Given the topic of the conference and the profile of the participants, the sample was convenient but purposefully selected. Although the sample was not ideal for calculating metric indicators such as construct validity and reliability, it was of high quality for determining the ranking of the challenges in using acquaintances among the offered occupations, as it included key and representative stakeholders working with complex patients in the Republic of Croatia and represents a relevant group for researching social capital in this context.

### 2.4. Analysis

Based on participants’ responses, three additive indices of social capital were constructed. Each occupation on the list carried a certain number of points, determined by the median of its position in the ranking of the difficulty of using acquaintances to obtain assistance. The ranking was created based on the average ratings of all participants in the second part of the questionnaire, where occupations were ordered from the least to the most challenging to mobilize in everyday work (for example, an occupation that most participants considered the most difficult to mobilize received the highest point coefficient).

The index for each type of social capital (perceived, potential, activated for patients) was calculated so that, for each occupation the respondent had in their network (depending on the type of capital), a corresponding number of points was assigned, and these points were then summed. For example, if a participant had the head nurse of a department (20 points), a lawyer (9 points), and a journalist (14 points) in their network, their perceived social capital amounted to 43 points (20 + 9 + 14). However, if the participant had activated only the contacts with the head nurse and the journalist for a patient, the activated social capital was 34 points (20 + 14). The difference between the indices enables an analysis of how well professionals are networked (perceived social capital), how strong these ties are (potential social capital), and whether they are already actively using that social capital in their work with patients (activated social capital for patients).

The use of the median instead of the mean ensures that extreme rankings by individual participants (for example, if one participant ranked a particular occupation extremely high or low) do not distort the point value. In this way, the points better correspond to the dominant perceptions of difficulty within the entire sample.

Descriptive statistics were used to describe the sociodemographic variables of the sample, including frequencies, percentages, arithmetic mean, median, and standard deviation. Continuous variables, which include all three social capital indices, years of work experience, and the number of workplaces changed, were analyzed using Spearman’s coefficient to assess linear relationships between them. Differences in social capital indices according to gender were tested using the independent samples *t*-test. The significance level for all analyses was set at α = 0.05. The analysis was performed using IBM SPSS 26, and the results are presented in the form of tables and graphical displays for easier interpretation.

### 2.5. Ethical Principles

The research was conducted in accordance with the ethical principles of scientific research, which include confidentiality, privacy, and anonymity of participant data. Participants were informed about the purpose of the research and its benefits and risks, and voluntarily agreed to participate in the study.

The research was approved by the Ethics Committee of the University of Zagreb School of Medicine on 27 March 2023 (Reference number: 380-59-10106-23-111/38, Class: 641-01/23-02/01).

## 3. Results

Out of a total of 74 conference participants, 18 were representatives of the organizational board and members of the World Bank team, who acted as observers and passive participants during the conference. Thirty-three individuals fully completed the survey questionnaire, representing a response rate of 58.92%.

Table 1 presents the basic sociodemographic characteristics of the respondents, including gender, years of work experience, number of workplaces changed, and profession. The majority of the sample were women (87.9%), while men accounted for 12.1% of respondents. Work experience ranged from a minimum of 4 to a maximum of 45 years, with an average of 24.03 years (SD = 10.39). The number of workplaces changed ranged from 2 to 10, with an average of 3.88 changes (SD = 1.867). Respondents self-reported their profession, and open-ended responses were subsequently grouped into three categories. The sample included 9 (27.3%) physicians regardless of specialization status, 18 (54.5%) nurses, and 6 (18.2%) respondents classified as “Other,” which included two social workers, a kinesiologist, a lawyer, an economist, and an occupational therapist.

Table 2 shows the ranking of various occupations according to the difficulty of using personal contacts to obtain help or services, with higher-ranked occupations considered to have greater influence or power. The higher the median value, the more “difficult” that connection is considered to be to utilize. The greatest challenge in using personal connections for obtaining help or services was perceived by respondents for representatives of local authorities, including prominent members of local communities, and representatives of the County Health Departments (mean = 22, M = 25, SD = 8), as well as for representatives of state authorities, such as employees in ministries (mean = 21, M = 23, SD = 10). Among occupations directly related to the healthcare system, the position of general practitioner was identified as the most challenging, followed by hospital department physician, emergency department physician, and head nurse of a hospital department. On the other hand, the least challenging occupations for using personal contacts were home support worker for older adults (mean = 11, M = 10, SD = 7), medical equipment and aids salesperson (mean = 11, M = 10, SD = 8), and priest (mean = 11, M = 10, SD = 8).

In Table 3, for each position, the percentages of respondents who reported having a personal contact with someone in that occupation (perceived social capital), who could request help or a service for themselves based on that contact (potential social capital), and who have used that contact to request help or a service for their patient in the past year (activated social capital for the patient) are shown. The occupations are ordered by the difficulty of utilizing connections, as determined in Table 2. The highest proportion of respondents reported having personal contact with the head nurse of a hospital department (72.7%), a community nurse (69.7%), a nurse in a mobile palliative care team (69.7%), a hospital department physician (69.7%), and a general practitioner (69.7%). In contrast, the lowest number of respondents had contacts with a home support worker for older adults (18.2%) and persons assisting with home care and patient care who are paid by the user (e.g., home help) (21.2%).

Regarding potential social capital, the largest number of respondents indicated the possibility of seeking help from a hospital department physician (72.7%), an emergency department physician (66.7%), and a general practitioner (63.6%), while the lowest potential for activating connections was recognized for home support worker for older adults (27.3%) and paid home help (24.2%).

In the past year, for their patients, the highest number of respondents activated contacts with a hospital department physician (54.5%), a general practitioner (45.5%), and the head nurse of a hospital department (45.5%). The least activated contacts were those with home support workers for older adults (12.1%) and paid home help (12.1%).

**Figure 1:**
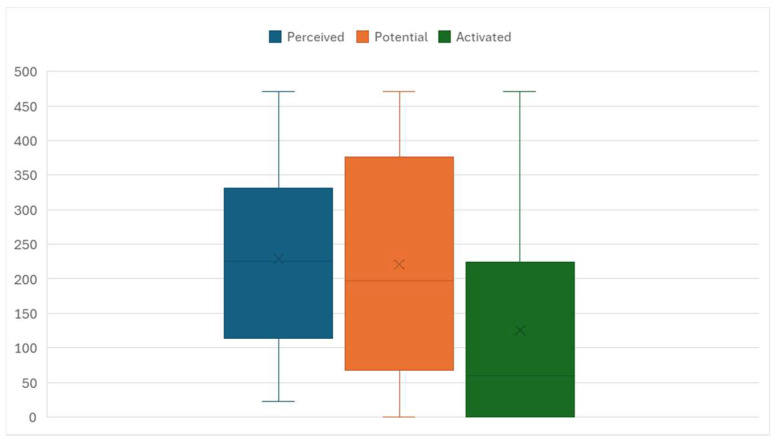
Descriptive indicators of social capital indices.

Figure 1 graphically presents the indices of perceived, potential, and activated social capital for patients. The colored area (rectangle) represents the middle 50% of values, that is, the interquartile range. The cross (X) marks the median, while the line within the colored area indicates the arithmetic mean. The results show that the index of perceived social capital had the highest median value (M = 258) and somewhat lower dispersion than the other indices (SD = 131.877). The potential social capital index had a slightly lower median (M = 274) and greater dispersion (SD = 152.101), while the activated social capital index for patients had the lowest median value (M = 98, SD = 152.381). Since some respondents clearly did not use social capital at all to help patients, for a portion of respondents, the activated social capital index shows a value of zero.

Table 4 presents the inter-item association matrix of sociodemographic variables and the three social capital indices. The results show that the three social capital indices are statistically significantly positively associated with each other (ρ = 0.595 to ρ = 0.533; *p* < 0.01), but the strength of the association is moderate. The number of workplaces was not statistically significantly associated with any of the social capital indices, while years of work experience was positively associated with the social capital index activated for patients (ρ = 0.374; *p* = 0.032).

## 4. Discussion

### 4.1. Methodological Contribution of the Instrument for Measuring Social Capital in the Care of Complex Patients

Recognizing and measuring social capital among professionals caring for complex patients is crucial for understanding how informal networks influence access, coordination, and continuity of care. Our research, utilizing an instrument that distinguishes between perceived, potential, and activated social capital for the patient, demonstrates that social capital is not a static network of acquaintances but a dynamic and multidimensional resource that is mobilized differently in everyday practice. The instrument, based on the position generator method, enables simultaneous measurement of the breadth of the social network, the functional strength of ties, and their actual activation for the needs of patients. This approach goes beyond the traditional understanding of social capital as mere “knowing someone” and introduces a key distinction between the existence of a connection and its concrete mobilization for the patient’s benefit. The multidimensional approach to measuring social capital, which includes the level of activation for the specific needs of patients, represents a significant advancement over previous instruments, which were mostly limited to the organizational context or to measuring social capital through perception or attitude [25].

The results show that all three dimensions of social capital are significantly interrelated, but also distinct from one another (ρ = 0.595 to ρ = 0.533; *p* < 0.01). This statistical association confirms the multidimensional nature of social capital, indicating that it cannot be viewed as a single, homogeneous phenomenon, but rather as a set of different yet connected elements. The highest median value is found for the perceived social capital index (M = 258), suggesting that professionals have broad networks of acquaintances within their work environment. However, the lower values of potential and activated social capital for patients point to a gap between having contacts and actually using them in practice. This gap illustrates the theory of “strong” and “weak” ties, according to which the existence of a connection does not guarantee its activation or functional use in specific situations [20].

It is particularly important to note that, in the dimension of activated social capital for the patient, there were also zero values recorded, further emphasizing the difference between possessing a network and its concrete mobilization for patient needs. Multidimensional measurement of social capital enables deeper insight into the cohesion and dynamics of professional relationships, helping to identify the strengths and weaknesses of the system. Understanding how these networks function in practice is extremely important for the development of sustainable and integrated models of care, especially in situations that require a high degree of coordination among stakeholders.

### 4.2. Implications for Healthcare Practice and System Development

Research shows that interprofessional collaboration is recognized as one of the key strategies for the successful transition of patients across different levels of care, especially for complex patient groups. Accordingly, ongoing coordination and formalization of cooperation between health and social care stakeholders are not only desirable practices but a necessity for ensuring quality, timely, and comprehensive care for complex patients [19,33,34]. Although this is recognized in relevant international and national publications, fragmentation of care due to inadequate cooperation remains a global challenge for modern health and long-term care systems. In their systematic review, Bolton, Logan, and Gittell examined the evidence base for the relational coordination theory by comprehensively analyzing all empirical studies on the predictors and outcomes of relational coordination [35]. Their findings highlight that, despite the theory’s strong theoretical foundation, its practical application across sectors remains highly challenging. Interprofessional collaboration is also linked to important dimensions such as job satisfaction [36,37] and perceived quality of care [38,39]. Although these dimensions were not the subject of our research, their connection further highlights the need to measure social networks and then systematically strengthen and formalize them.

In addition to its contribution to the quality and integration of care, social capital may also have important implications for system efficiency and cost reduction, particularly when considered within the broader framework of integrated care development. Research suggests a potential for cost savings through integrated care, although the overall evidence remains limited. Better coordination in the delivery of care can help reduce the length of hospital stays, prevent unnecessary emergency admissions, and minimize complications caused by gaps in care continuity [40,41,42,43]. These outcomes are frequently associated with stronger coordination and collaboration among professionals, dimensions in which social capital plays a significant role. Accordingly, bridging social capital has a significant role as an enabler of professional integration, as it supports collaboration among professionals both within and across institutions. However, over-reliance on informal networks may hinder the development of structured, sustainable integration models. In this regard, social capital should be recognized not as a substitute for formal integration mechanisms, but as a foundation upon which system-level coordination can be built.

The instrument developed for this study was applied in a model of care for complex patients that includes a wide range of professionals from the health, social care, and community systems [19,33]. One important feature of the instrument is its ability to rank professional roles according to the challenges in using professional connections in the context of care for complex patients. This dimension of the instrument allows for its adaptation and application in various social, organizational, and temporal contexts, including its potential use in other countries and health systems. It is important to note that such rankings reflect existing hierarchical structures within the system and the perceived importance and participation of certain professions/professionals in patient care, which can affect the availability and activation of certain connections. Furthermore, professionals’ attitudes toward care coordination and specific roles in the system significantly influence the further development and implementation of interprofessional networks and care integration. Power struggles in this domain have already been described in the literature [10,43].

For example, in our results, the most frequently activated contacts were the hospital department physician (54.5%), general practitioner (45.5%), and head nurse of a hospital department (45.5%), implying their important roles in planning and delivering care at various levels. Their frequent interaction with patients and other professionals facilitates the creation and maintenance of functional networks, enabling a faster and more efficient response to patient needs. In contrast, occupations such as home support worker for older adults and paid home help recorded the lowest levels of perceived, potential, and activated social capital for the patient, which may indicate their weaker integration into broader professional networks and lower recognition of their role, despite the importance they have in daily care and patient support [44,45]. Lay home support assistants are noted as informal caregivers in certain cultures, which gives them the potential to further leverage their role [46].

Highly ranked representatives of local and state authorities, despite their relatively low activation in care networks for complex patients, are perceived as the most challenging to use for personal connections to obtain help or services. This may imply their high social and institutional power, which often involves more complex procedures and more formal channels of communication. Such status can limit accessibility and flexibility in activating their connections, indicating a functional difference between strategic stakeholders with higher authority and operational professionals who are more frequently involved in direct care coordination.

In the context of involving stakeholders outside the healthcare system in the care of complex patients, our results show that non-health professionals, such as social workers, priests, journalists, and police officers, are ranked lower in terms of perceived, potential, and activated social capital for the patient. This finding may reflect their occasional and targeted involvement in the care process, but possibly also the fact that their integration into the care network most often occurs through clearly defined or institutionalized coordination mechanisms, so there is no need to activate social capital when cooperation is established.

On the one hand, well-developed personal networks enable professionals to expedite access to services, facilitate coordination between different sectors, and ensure timely, integrated care for complex patients. This function of social capital is particularly evident in the context of bridging social capital, where connecting different professionals and sectors allows for a more flexible and effective response to the complex needs of patients. In our study, the most frequently activated contacts were the hospital department physician, general practitioner, and head nurse of a hospital department, confirming the central role of these stakeholders in daily care coordination and the building of functional networks. On the other hand, excessive reliance on such networks can deepen inequalities and increase system vulnerability, especially for professionals who do not have access to strong informal connections. The presence of zero values in activated social capital shows that not all professionals are equally able or willing to use their networks for the benefit of patients, which can lead to uneven care outcomes and dependence on individual relationships.

The results show that professionals with longer work experience have more developed networks and a greater ability to activate these connections (positive association ρ = 0.374; *p* = 0.032), indicating that experience not only expands the network of contacts but also increases the skill of mobilizing them. Although this is generally perceived as an advantage, it can also be potentially risky, as there may be over-reliance on networks, sometimes bypassing formal mechanisms. This highlights the need to support less experienced professionals to ensure equal access to support and resources in the care of complex patients. Additionally, the system becomes vulnerable to staff changes; when key professionals leave the system, their networks leave with them, potentially creating significant gaps in care and coordination.

This dynamic underscores the importance of assessing the strength and vulnerability of a system that relies heavily on the individual social capital of professionals, rather than on established protocols and procedures. A system that predominantly depends on informal networks can increase inequalities in care, as the availability and quality of services become dependent on individual networks and the capacities of specific professionals, which may jeopardize the continuity and timeliness of care.

### 4.3. Strengths and Limitations

Despite certain methodological limitations, the applied instrument for measuring social capital demonstrated several important strengths that confirm its scientific value and potential for broader application in other contexts. One of the main strengths of the instrument lies in its ability to rank professional roles according to the challenge of utilizing professional connections in the context of complex patient care. This ranking capability makes the instrument flexible and adaptable to different social, organizational, and temporal settings, including other countries and healthcare systems.

It is particularly important to emphasize that, although the number of participants was relatively limited, the sample consisted of highly specialized experts with practical experience in working with complex patients, who form the backbone of the complex care system in Croatia.

On the other hand, the main limitation of the study is the sample size. While preliminary results indicate interconnections among the observed indicators and confirm the basic hypotheses, further testing of the instrument’s reliability and construct validity is necessary to ensure its scientific robustness. Therefore, the results should be interpreted as an introduction to further research, rather than as definitive generalizations.

Although full psychometric validation was beyond the scope of this pilot study, the findings provide initial empirical insights into dimensions of social capital that have not previously been measured in this context. These results provide a valuable foundation for further refinement of the instrument and support its potential relevance in future research and practice.

### 4.4. Future Research and Application of the Social Capital Measurement Instrument

Based on the results and identified limitations of this study, the following recommendations are proposed as guidelines for future research and practical application of the social capital measurement instrument in the context of care for complex patients.

First, it is recommended to expand the methodological approach by conducting research on a larger sample of participants. A larger sample would provide a stronger statistical basis for analysis and increase the external validity of the results, thereby enabling the generalization of findings to a broader population of health and social care professionals. This is particularly important for further testing the robustness and sensitivity of the instrument to variations across different settings and professional groups.

Additionally, further validation procedures for the instrument are essential. Specifically, it is recommended to examine construct validity to ensure that the instrument accurately measures the theoretically predicted dimensions of social capital. In parallel, the reliability of the instrument should be assessed, including test–retest methods and internal consistency (e.g., using Cronbach’s alpha coefficient), to confirm the stability of results over time.

Operationally, for future research within the Croatian context, it is advisable to utilize the established median ranking of professional roles according to the challenge of leveraging professional connections, as determined in this sample. Given the high level of expertise and experience among participants, this ranking can be considered representative of professionals currently involved in the care of complex patients in Croatia. This approach may significantly shorten the length of the questionnaire in future studies and facilitate its use, eliminating the need to repeatedly collect data on the ranking of professions in each survey administration.

Finally, a deeper analysis of differences among professions regarding perceived, potential, and activated social capital for patients is recommended. Such analysis could contribute to a more precise understanding of the roles of specific groups within the care network and support the development of targeted interventions to strengthen interprofessional collaboration and the integration of less recognized stakeholders, such as home care workers or social workers.

In addition to methodological considerations, the findings also suggest directions for practical and policy-related action. In settings where strong social capital among professionals is already present, such informal networks should be recognized as strategic assets for enhancing care coordination. These networks may serve as a foundation for the formalization of collaboration structures, especially if supported through institutional recognition and the inclusion of additional stakeholders.

Good practices developed through informal professional connections can be identified and used as a starting point for systematic integration, turning isolated efforts into sustainable, interprofessional cooperation. This approach is particularly relevant for health systems where formal mechanisms for integration remain under development or are in the early stages of implementation.

The instrument developed in this study may also assist decision-makers in mapping gaps and strengths within professional networks, enabling more targeted investments in capacity building and more effective allocation of resources. In healthcare systems similar to Croatia’s, which are undergoing a transition toward the development of integrated long-term care, this represents a pragmatic, scalable, and context-appropriate pathway for building resilient, inclusive, and patient-centered care coordination mechanisms.

## 5. Conclusions

This pilot study confirmed the importance and presence of social capital among professionals involved in the care of complex patients, highlighting the need for its precise measurement and a deeper understanding of it within the context of care.

The developed instrument, based on the position generator method and expanded to assess perceived, potential, and activated social capital for the patient, enabled a differentiated view of the distinction between the existence of professional networks and their actual use for the benefit of patients. The instrument proved to be a suitable tool for mapping and understanding professional connections within the care system for complex patients.

Accumulated work experience of professionals contributes to a greater ability to mobilize professional networks, whereas formal changes in the work environment alone are insufficient to enhance the activation of social capital. Therefore, it is important to have instruments capable of comprehensively assessing the potential and possible significance of social capital in various healthcare settings.

Given the need to develop a coordinated care system for complex patients and the implications that professionals’ social capital has for the development of such a system, this pilot study lays the foundation for future research with a larger sample, aiming at further validation of the instrument.

## Figures and Tables

**Table 1:** Sociodemographic characteristics of respondents.

	N (%)
**Gender**	
Male	4 (12.1)
Female	29 (87.9)
**Years of work experience**	
Mean	24.03
SD	10.39
Minimum	4
Maximum	45
**Number of workplaces changed**	
Mean	3.88
SD	1.867
Minimum	2
Maximum	10
**Profession**	
Physician	9 (27.3)
Nurse	18 (54.5)
Other	6 (18.2)

**Table 2:** Ranking by the difficulty of utilizing connections.

	Mean	Median ^1^	SD
Representative of local authorities—prominent member of the local community	22	25	8
Representative of local authorities (e.g., head of the County Health Department)	22	25	8
Representative of state authorities (e.g., ministry employee)	21	23	10
General practitioner	20	22	7
Hospital department physician	19	21	9
Head nurse of a hospital department	19	20	8
Emergency department physician	19	20	8
Physician in a mobile palliative care team	17	18	7
Pharmacist	16	18	8
Hospital department nurse	16	16	8
Hospital discharge planning nurse	16	16	9
Visiting/community nurse	16	16	7
Nurse in a mobile palliative care team	16	16	8
Nurse/technician in the emergency department	15	15	8
Nurse in a general practitioner team	15	15	8
Nurse/technician in the outpatient emergency medical services team	14	15	7
Palliative care coordinator	15	15	9
Outpatient emergency medical services physician	16	15	8
Home care nurse	14	14	8
Home care physiotherapist	14	14	7
Social worker	16	14	9
Journalist	14	14	9
Representative of volunteer associations or volunteer	13	13	9
Police officer	12	12	8
Home support worker for older adults	11	10	7
Medical equipment and aids salesperson	11	10	8
Priest	11	10	8
Lawyer	12	9	9
Persons who assist with home care and patient care, paid by the user (e.g., home help)	12	9	9
Handyman for minor repairs	12	9	10

^1^ Ordered by median from the most challenging positions for utilizing connections to the least challenging positions.

**Table 3:** Perceived, potential, and activated social capital.

	Perceived ^1^		Potential ^2^		Activated ^3^	
	N	%	N	%	N	%
Representative of local authorities—prominent member of the local community	14	42.4	20	60.6	10	30.3
Representative of local authorities (e.g., head of the county Department of Health)	16	48.5	14	42.4	7	21.2
Representative of state authorities (e.g., ministry employee)	14	42.4	14	42.4	6	18.2
General practitioner	23	69.7	21	63.6	15	45.5
Hospital department physician	23	69.7	24	72.7	18	54.5
Head nurse of a hospital department	24	72.7	20	60.6	15	45.5
Emergency department physician	18	54.5	22	66.7	13	39.4
Physician in a mobile palliative care team	15	45.5	16	48.5	10	30.3
Pharmacist	18	54.5	14	42.4	11	33.3
Hospital department nurse	17	51.5	20	60.6	12	36.4
Hospital discharge planning nurse	18	54.5	15	45.5	10	30.3
Visiting/community nurse	23	69.7	20	60.6	14	42.4
Nurse in a mobile palliative care team	23	69.7	20	60.6	14	42.4
Nurse/technician in the emergency department	14	42.4	18	54.5	9	27.3
Nurse in a general practitioner team	21	63.6	20	60.6	14	42.4
Nurse/technician in the outpatient emergency medical services team	14	42.4	16	48.5	8	24.2
Palliative care coordinator	24	72.7	20	60.6	12	36.4
Outpatient emergency medical services physician	13	39.4	12	36.4	7	21.2
Home care nurse	20	60.6	18	54.5	9	27.3
Home care physiotherapist	12	36.4	12	36.4	8	24.2
Social worker	16	48.5	15	45.5	12	36.4
Journalist	15	45.5	17	51.5	5	15.2
Representative of volunteer associations or volunteer	15	45.5	19	57.6	8	24.2
Police officer	12	36.4	12	36.4	5	15.2
Home support worker for older adults	6	18.2	9	27.3	4	12.1
Medical equipment and aids salesperson	19	57.6	20	60.6	13	39.4
Priest	16	48.5	14	42.4	5	15.2
Lawyer	16	48.5	16	48.5	7	21.2
Persons who assist with home care and patient care, paid by the user (e.g., home help)	7	21.2	8	24.2	4	12.1
Handyman for minor repairs	20	60.6	13	39.4	7	21.2

^1^ Perceived social capital refers to respondents who have personal contact with someone in that occupation. ^2^ Potential social capital—respondents who could request help or a service for themselves based on that contact. ^3^ Activated social capital for the patient—respondents who have used that contact to request help or a service for their patient.

**Table 4:** Inter-item association matrix of sociodemographic variables and the three (indices of) forms of social capital (Spearman’s ρ).

	1	2	3	4
1. Number of workplaces changed during career	1			
2. Years of work experience	0.440 *	1		
3. Perceived social capital	0.12	0.137	1	
4. Potential social capital	0.234	0.321	0.595 **	1
5. Activated social capital for the patient	0.252	0.374 *	0.533 **	0.595 **

* *p* < 0.05; ** *p* < 0.01.

## Data Availability

Data is available from the corresponding author upon a reasonable request.

## Appendix A

Questionnaire.

Dear Participant,

As part of the project “Improving Care for Complex Patients” at the School of Medicine University of Zagreb, we are conducting research aimed at testing a new instrument for measuring selected elements of social capital in healthcare, with a special emphasis on complex and long-term care. Social capital in healthcare refers to networks, norms, and social support that can significantly influence the effectiveness of care provision.

Your participation in this survey will help us understand these aspects in the context of long-term care. All data collected through this survey are anonymous. Only the researchers involved in this project will have access to the data, and the results will be presented in aggregate form.

Participation in the survey is entirely voluntary.

The expected time to complete the survey is 10 min.

Principal Investigator: Full Professor Aleksandar Džakula (Center for Health Systems, Policies and Diplomy, School of Medicine, University of Zagreb)

Research team members: Dorja Vočanec, PhD; Marko Marelić, PhD and Maja Banadinović, MHA

For additional information, feel free to contact us by email: czspd@mef.hr.

Thank you in advance for your time and cooperation.

1. I hereby confirm that I have been informed about the objectives of this research and agree to participate.
  - I agree
  - I do not agree
2. Below is a list of professions. For each profession, please indicate whether you have a personal contact in that profession, whether you could request help or a service for yourself based on that personal contact, and whether you have used that personal contact in the past year to request help or a service for your patient.
  	**I Have a Personal Contact**	**I Could Request Help or a Service for Myself Based on This Contact**	**In the Past Year, I Have Requested Help or a Service for My Patient Through This Contact**
  Head nurse of a hospital department			
  Hospital department nurse			
  Nurse/technician in the emergency department			
  Hospital discharge planning nurse			
  Visiting/community nurse			
  Nurse in a general practitioner team			
  Nurse/technician in the outpatient emergency medical services team			
  Nurse in a mobile palliative care team			
  Home care nurse			
  Palliative care coordinator			
  Hospital department physician			
  Emergency department physician			
  General practitioner			
  Physician in a mobile palliative care team			
  Outpatient emergency medical services physician			
  Pharmacist			
  Home care physiotherapist			
  Home support worker for older adults			
  Medical equipment and aids salesperson			
  Social worker			
  Lawyer			
  Representative of volunteer associations or volunteer			
  Persons who assist with home care and patient care, paid by the user (e.g., home help)			
  Representative of local authorities—prominent member of the local community			
  Representative of local authorities (e.g., head of the county Department of Health)			
  Representative of state authorities (e.g., ministry employee)			
  Handyman for minor repairs			
  Priest			
  Journalist			
  Police officer			
3. Please rank the offered professions according to how challenging it is to use personal contacts to obtain help or services in those professions, with higher-ranked professions being considered as having more influence or power.
  ⮚ Head nurse of a hospital department
  ⮚ Hospital department nurse
  ⮚ Nurse/technician in the emergency department
  ⮚ Hospital discharge planning nurse
  ⮚ Visiting/community nurse
  ⮚ Nurse in a general practitioner team
  ⮚ Nurse/technician in the outpatient emergency medical services team
  ⮚ Nurse in a mobile palliative care team
  ⮚ Home care nurse
  ⮚ Palliative care coordinator
  ⮚ Hospital department physician
  ⮚ Emergency department physician
  ⮚ General practitioner
  ⮚ Physician in a mobile palliative care team
  ⮚ Outpatient emergency medical services physician
  ⮚ Pharmacist
  ⮚ Home care physiotherapist
  ⮚ Home support worker for older adults
  ⮚ Medical equipment and aids salesperson
  ⮚ Social worker
  ⮚ Lawyer
  ⮚ Representative of volunteer associations or volunteer
  ⮚ Persons who assist with home care and patient care, paid by the user (e.g., home help)
  ⮚ Representative of local authorities—prominent member of the local community
  ⮚ Representative of local authorities (e.g., head of the county Department of Health)
  ⮚ Representative of state authorities (e.g., ministry employee)
  ⮚ Handyman for minor repairs
  ⮚ Priest
  ⮚ Journalist
  ⮚ Police officer
4. Sex
  - Male
  - Female
5. How many years of work experience do you have? _________
6. Profession (e.g., master of nursing, specialist in family medicine) _________
7. Occupation (e.g., palliative care coordinator, community nurse, general practitioner) _________
8. How many workplaces have you changed during your career so far? ________

## References

[1] Framework for Countries to Achieve an Integrated Continuum of Long-Term Care. https://www.who.int/publications/i/item/9789240038844.

[2] People-Centred Health Care: A Policy Framework. https://www.who.int/publications/i/item/9789290613176.

[3] Nicolaus S., Crelier B., Donzé J.D., Aubert C.E. (2022). Definition of Patient Complexity in Adults: A Narrative Review. J. Multimorb. Comorbidity.

[4] Fortin M., Bravo G., Hudon C., Vanasse A., Lapointe L. (2005). Prevalence of Multimorbidity among Adults Seen in Family Practice. Ann. Fam. Med..

[5] Lehnert T., Heider D., Leicht H., Heinrich S., Corrieri S., Luppa M., Riedel-Heller S., König H.-H. (2011). Review: Health Care Utilization and Costs of Elderly Persons with Multiple Chronic Conditions. Med. Care Res. Rev. MCRR.

[6] Manning E., Gagnon M. (2017). The Complex Patient: A Concept Clarification. Nurs. Health Sci..

[7] Džakula A., Lončarek K., Vočanec D. (2023). Complex Patients—An Academism or Reality?. Croat. Med. J..

[8] Strengthening the Integrated Delivery of Long-Term Care in the European Region. https://iris.who.int/bitstream/handle/10665/353912/WHO-EURO-2022-5330-45095-64318-eng.pdf.

[9] Varda D., Shoup J.A., Miller S. (2012). A Systematic Review of Collaboration and Network Research in the Public Affairs Literature: Implications for Public Health Practice and Research. Am. J. Public Health.

[10] Vočanec D., Lončarek K., Sović S., Džakula A. (2023). Nurse Coordinator of Care as a Facilitator of Integration Processes in Palliative Care. J. Clin. Nurs..

[11] Epstein J.A., Wu A.W. (2021). Delivering Complex Care: Designing for Patients and Physicians. J. Gen. Intern. Med..

[12] Health at a Glance: Europe 2024. https://www.oecd.org/en/publications/health-at-a-glance-europe-2024_b3704e14-en.html.

[13] European Commission A European Care Strategy for Caregivers and Care Receivers. https://employment-social-affairs.ec.europa.eu/news/european-care-strategy-caregivers-and-care-receivers-2022-09-07_en.

[14] Ministry of Health of the Republic of Croatia National Health Development Plan for the Period from 2021 to 2027. https://zdravlje.gov.hr/UserDocsImages/2022%20Objave/Nacionalni%20plan%20razvoja%20zdravstva%202021.-2027..pdf.

[15] Republic of Croatia Health Care Act. https://www.zakon.hr/z/190/zakon-o-zdravstvenoj-zastiti.

[16] Ministry of Health of the Republic of Croatia Plan and Programme of Health Care Measures. https://narodne-novine.nn.hr/clanci/sluzbeni/2023_10_127_1773.html.

[17] Džakula A., Vočanec D., Relić D. (2023). From Fragmented Care Back to Social Medicine: European Policy Responses to the Needs of Complex Patients. Croat. Med. J..

[18] Whitt N., Harvey R., McLeod G., Child S. (2007). How Many Health Professionals Does a Patient See during an Average Hospital Stay?. N. Z. Med. J..

[19] Tahan H.A. (2007). One Patient, Numerous Healthcare Providers, and Multiple Care Settings: Addressing the Concerns of Care Transitions through Case Management. Prof. Case Manag..

[20] Field J. (2008). Social Capital.

[21] Moore S., Kawachi I. (2017). Twenty Years of Social Capital and Health Research: A Glossary. J. Epidemiol. Community Health.

[22] Putnam R.D., Leonardi R., Nanetti R.Y. (1994). Making Democracy Work: Civic Traditions in Modern Italy.

[23] Derose K.P., Varda D.M. (2009). Social Capital and Health Care Access: A Systematic Review. Med. Care Res. Rev. MCRR.

[24] Villalonga-Olives E., Kawachi I. (2017). The Dark Side of Social Capital: A Systematic Review of the Negative Health Effects of Social Capital. Soc. Sci. Med..

[25] Ansmann L., Hower K.I., Wirtz M.A., Kowalski C., Ernstmann N., McKee L., Pfaff H. (2020). Measuring Social Capital of Healthcare Organizations Reported by Employees for Creating Positive Workplaces—Validation of the SOCAPO-E Instrument. BMC Health Serv. Res..

[26] Sheingold B.H., Sheingold S.H. (2013). Using a Social Capital Framework to Enhance Measurement of the Nursing Work Environment. J. Nurs. Manag..

[27] Kouvonen A., Kivimäki M., Vahtera J., Oksanen T., Elovainio M., Cox T., Virtanen M., Pentti J., Cox S.J., Wilkinson R.G. (2006). Psychometric Evaluation of a Short Measure of Social Capital at Work. BMC Public Health.

[28] Shiell A., Hawe P., Kavanagh S. (2020). Evidence Suggests a Need to Rethink Social Capital and Social Capital Interventions. Soc. Sci. Med..

[29] Verhaeghe P.-P., Li Y., Li Y. (2015). The Position Generator Approach to Social Capital Research: Measurements and Results. Handbook of Research Methods and Applications in Social Capital.

[30] Konferencija o Unaprjeđenju Skrbi za Kompleksne Pacijente u Biogradu. https://ezadar.net.hr/dogadaji/4358401/konferencija-o-unaprjedjenju-skrbi-za-kompleksne-pacijente-u-biogradu/.

[31] Druga Konferencija o Unaprjeđenju Skrbi za Kompleksne Pacijente: Mogu li Dugotrajna Skrb i Kompleksni Pacijenti Buditi Optimizam?. https://klikni.news/aktualno-news/2024/05/30/druga-konferencija-o-unaprjedenju-skrbi-za-kompleksne-pacijente-mogu-li-dugotrajna-skrb-i-kompleksni-pacijenti-buditi-optimizam/.

[32] Rupasinghe Y.N., Schack M.V., Bolongaita S.A., Džakula A. An Overview of Long-Term Care Challenges and Opportunities (English).

[33] Geese F., Schmitt K.-U. (2023). Interprofessional Collaboration in Complex Patient Care Transition: A Qualitative Multi-Perspective Analysis. Healthcare.

[34] Reeves S., Pelone F., Harrison R., Goldman J., Zwarenstein M. (2017). Interprofessional Collaboration to Improve Professional Practice and Healthcare Outcomes. Cochrane Database Syst. Rev..

[35] Bolton R., Logan C., Gittell J.H. (2021). Revisiting Relational Coordination: A Systematic Review. J. Appl. Behav. Sci..

[36] Wiedermann C.J., Barbieri V., Engl A., Piccoliori G. (2024). Impact of Relational Coordination on Job Satisfaction and Willingness to Stay: A Cross-Sectional Survey of Healthcare Professionals in South Tyrol, Italy. Behav. Sci..

[37] Zhang H., Sun L., Zhang Q. (2022). How Workplace Social Capital Affects Turnover Intention: The Mediating Role of Job Satisfaction and Burnout. Int. J. Environ. Res. Public Health.

[38] Pedersen L.M., Jakobsen A.L., Buttenschøn H.N., Haagerup A. (2023). Positive Association between Social Capital and the Quality of Health Care Service: A Cross-Sectional Study. Int. J. Nurs. Stud..

[39] Chang W.-Y., Ma J.-C., Chiu H.-T., Lin K.-C., Lee P.-H. (2009). Job Satisfaction and Perceptions of Quality of Patient Care, Collaboration and Teamwork in Acute Care Hospitals. J. Adv. Nurs..

[40] Valentijn P.P. (2016). Rainbow of Chaos: A Study into the Theory and Practice of Integrated Primary Care. Int. J. Integr. Care.

[41] Rocks S., Berntson D., Gil-Salmerón A., Kadu M., Ehrenberg N., Stein V., Tsiachristas A. (2020). Cost and Effects of Integrated Care: A Systematic Literature Review and Meta-Analysis. Eur. J. Health Econ..

[42] Nolte E., Pitchforth E. (2014). What Is the Evidence on the Economic Impacts of Integrated Care?.

[43] Hall P. (2005). Interprofessional Teamwork: Professional Cultures as Barriers. J. Interprof. Care.

[44] Genet N., Boerma W.G., Kringos D.S., Bouman A., Francke A.L., Fagerström C., Melchiorre M.G., Greco C., Devillé W. (2011). Home Care in Europe: A Systematic Literature Review. BMC Health Serv. Res..

[45] The Challenging Roles of Informal Carers—UNECE Policy Brief on Ageing No. 22. https://www.decadeofhealthyageing.org/find-knowledge/resources/publications/the-challenging-roles-of-informal-carers---unece-policy-brief-on-ageing-no.-22.

[46] Afram B., Verbeek H., Bleijlevens M.H.C., Hamers J.P.H. (2015). Needs of Informal Caregivers during Transition from Home towards Institutional Care in Dementia: A Systematic Review of Qualitative Studies. Int. Psychogeriatr..

